# Use of ICG imaging to confirm bowel viability after upper mesenteric stenting in patient with acute mesenteric ischemia: Case report

**DOI:** 10.1016/j.ijscr.2019.07.077

**Published:** 2019-07-31

**Authors:** Khitaryan Аlexander, Miziev Ismail, Murlychev Alexander, Taranov Ivan, Voronova Olga, Shatov Dmitry, Golovina Anastasiya, Melnikov Denis

**Affiliations:** a NGHCI Railway Clinical Hospital at the “Rostov-Glavnyy” Station, OAO Russian Railways, Varfolomeeva Street 92, Rostov-on-Don, Russian Federation; b FSBEI HE Rostov State Medical University of the Ministry of Health of the Russian Federation, Nakhichevansky Lane 19, Rostov-on-Don, Russian Federation; c FSBEI HE Kabardino-Balkarian State University named after Berbekov H.M., Chernyshevskiy Street 173, Nalchik, Russian Federation; d SBI of Rostov region "Bureau of Forensic Medical Examination", Bodraya Street 88/35, Rostov-on-Don, Russian Federation

**Keywords:** Acute mesenteric ischemia, Endovascular intervention, Indocyanine green imaging, Case report

## Abstract

**Introduction:**

Acute mesenteric ischemia (AMI) is a rare and life-threatening disease. Despite modern advances in open and endovascular treatments it has unacceptably high mortality rate.

**Presentation of case:**

A 70-year-old man with NSTEMI was diagnosed with acute mesenteric ischemia on the 3rd day after primary coronary intervention. CT with intravenous contrast revealed a generalized abdominal aorta atherosclerosis and a circular thrombus in SMA. Then a superior mesenteric angiography with aspiration thrombectomy, percutaneous transluminal angioplasty and stenting was performed. The next day abdominal pain still persisted. A diagnostic laparoscopy with ICG fluorescence was implemented. There were no ischemia and necrotic changes. The patient was alive at 4 months after the operative treatment of AMI.

**Discussion:**

In patients with myocardial infarction, atrial fibrillation, accompanied by any invasive procedure, especially endovascular, the incidence of thrombosis and thromboembolic complications is high. This is the key to the suspicion of such a serious complication as AMI. It can be assumed that in some cases, with adequate revascularization of the small intestine, reperfusion syndrome may occur and cause ischemic necrosis of the intestinal wall. In this case it is necessary to perform control laparoscopy in 24–48 h.

**Conclusion:**

In patients with suspected AMI timely applying of MDCT, angiography, endovascular revascularization and ICG quality control of perfusion after revascularization are expedient to improve the results of treatment. These patients should be treated by a multidisciplinary team consisting of a cardiologist, a cardiovascular and endovascular surgeon, a general surgeon with experience in working with such patients.

## Highlights

• A 70-year-old man with NSTEMI was diagnosed with AMI on the 3rd day after primary coronary intervention.
• Acute mesenteric ischemia is a life-threatening disease with high mortality rate, varying from 40% to 69%.
• The man underwent thoracoscopic SMA angiography with aspiration thrombectomy and stenting.
• On the next day a diagnostic laparoscopy with ICG fluorescence was implemented to access bowel viability.
• The patient is alive at 4 months after operative treatment of AMI.

## 1 Introduction

Acute mesenteric ischemia (AMI) is a rare and life-threatening disease that can cause mesenteric infarction, intestinal necrosis, overwhelming inflammatory response and death [1]. The incidence of AMI increases due to ageing of population and high rate of comorbidities, becoming a problem of current interest [2]. Despite modern advances in open and endovascular treatment options, it has unacceptably high mortality rate, varying from 40% to 69% [[3], [4], [5]]. Unfortunately, mesenteric ischemia has no specific signs or routine clinical tests and usually presents with non-specific abdominal pain, which can be caused by a large number of pathological conditions. Broad differential diagnosis leads to misdiagnosis and delay in appropriate treatment.

Early diagnosis and intervention are crucial for patient’s prognosis [[6], [7], [8]]. According to some research, mortality rate was 10.6% if patient was operated in the first 24 h after the onset of symptoms vs. 72.9% if operated after 24 h [7].

In this report we describe a survival case of AMI in which the patient underwent percutaneous superior mesenteric artery (SMA) angioplasty with stenting followed by laparoscopy with indocyanine green (ICG) fluorescence imaging to confirm sufficient bowel perfusion and viability.

The patient was treated in a non-governmental clinical hospital. The work was reported in line with the SCARE criteria [9].

## 2 Presentation of case

A 70-year-old man was admitted to internal medicine department with complaints of heartburn and intolerable chest pain with irradiation to the left arm and shoulder, neck and back. Pain had appeared a day before during domestic physical activity. Based on positive troponine blood test, electrocardiography (ECG), echocardiography and standard clinical diagnostic measures the patient was diagnosed with: Coronary artery disease, non-ST segment elevation myocardial infarction of front-side and apex; Killip class II; heart failure class IV (NYHA classification); Grace scale 174 scores. The patient underwent primary coronarography, balloon angioplasty and left circumflex artery stenting. The time between admission and primary percutaneous coronary intervention (PCI) was 70 min. The patient tolerated the intervention well and was transferred from the IC unit to the internal medicine department ward on the second day after the PCI.

On the 3^rd^ day after breakfast the patient complained of severe diffuse abdominal pain and diarrhea. Clinical examination showed abdominal distention, active peristalsis and no signs of peritoneal irritation. Antispasmodic and nonsteroidal anti-inflammatory drugs did not cease the pain. Blood tests revealed leukocytosis of 21.2 × 10*12/L, alanine transaminase 49 IU/L, aspartate transaminase 89 IU/L, alkaline phosphatase 207 IU/L, D-dimer level of 500 ng/ml (laboratory test dynamics see Table 1).

**Table 1:** Laboratory tests dynamics.

In-hospital day	Leukocytes, x 10*12/L	ALT, IU/L	AST, IU/L	D-dimer, ng/ml	Lactate, mmol/L	Procalcitonin, ng/ml
1	12,1	35	52	–	2,1	–
3	21,2	32	41	500	3,45	2,1
4	18,9	–	–	470	3,33	0,52
5	21,8	–	–	–	–	0,50
9	19,0	–	–	–	–	–
15	12,4	31	40	–	–	0,02

Abdominal ultrasound showed intense flatulence, no intraabdominal free fluid and no other pathology. Duplex scan was impossible due to poor visualization with bowel gas.

**Fig. 1:**
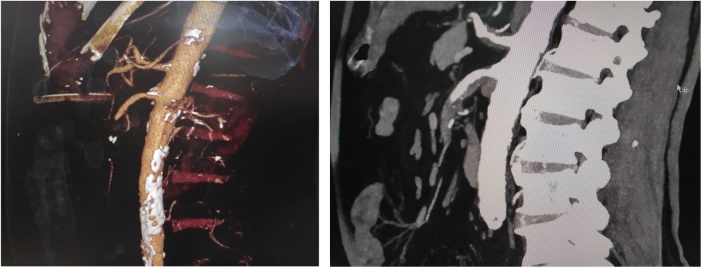
Computed tomography with intravenous contrast showing SMA occlusion.

In a view of this sudden diffuse continuous abdominal pain with no findings on clinical examination, patient’s anamnesis data (age, myocardial infarction followed by vascular manipulation, hypertension, dyslipidemia) AMI was suspected. Then computed tomography with intravenous contrast was performed (Fig. 1). It revealed CT-signs of generalized abdominal aorta atherosclerosis and circular thrombus with 30 mm length in superior mesenteric artery right below its takeoff from the aorta. Small intestine was dilatated with horizontal liquid levels.

The decision to perform superior mesenteric angiography with aspiration thrombectomy, percutaneous transluminal angioplasty and stenting was made. The time between abdominal pain onset and endovascular intervention was 8 h.

**Fig. 2:**
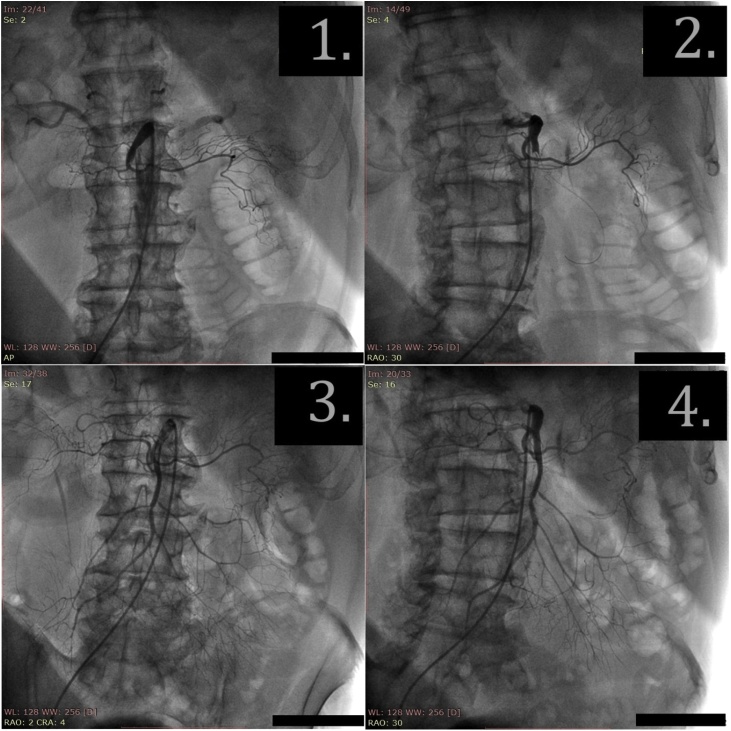
The angiograms of SMA. (2.1). Pretreatment angiogram: occlusion of SMA. (2.2). The guidewire is advanced behind the occlusion. (2.3–2.4). After thrombus aspiration and stenting; bloodflow in SMA is restored.

**Fig. 3:**
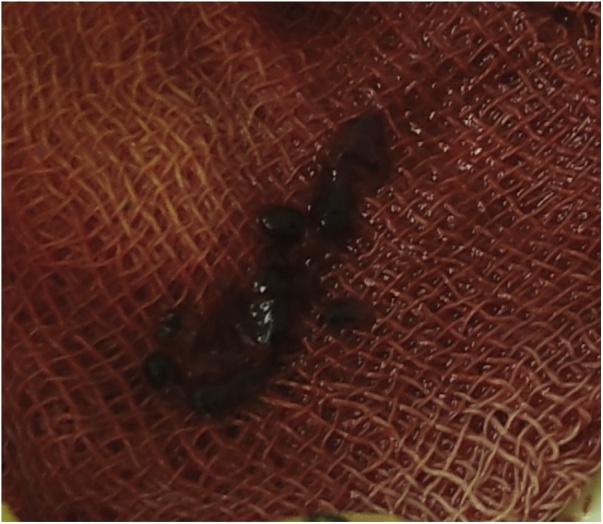
The specimen-aspired thrombus.

Under local anesthesia with 0.5% lidocaine solution (5 ml), JR-4 6 F guide catheter (Vista Bright Tip, Cordis) was installed to the SMA through right femoral approach. The angiogram was performed showing proximal SMA occlusion in 35 mm from its arising (Fig. 2 (2.1)). 10,000 IU of heparin were administered intra-arterial. The guidewire (Hi-Torque Whisper ES, Abbott) was inserted through the occlusion into the distal segment of SMA (Fig. 2 (2.2)). Thrombus was aspired with extraction catheter (QuickCat, Spectranetics), and blood clots were removed (Fig. 3). During a control angiography, SMA was permeable; there was a stenosis and dissection of the artery in the proximal third.

The coronary stent with a drug coating (3.5 × 23 mm, Abbott Xience) was positioned and expanded under the pressure of 14 atm to the dissection zone of the proximal part of the SMA. The control angiography showed a satisfactory result; residual stenosis and vessel dissection were not detected (Fig. 2 (2.3), (2.4)). The guidewire and catheter were removed; introducer was left until hemostasis was normalized. In total 200 ml of Ultravist contrast were introduced.

Then the patient was transferred to ICU. The next day (4th in-hospital day) abdominal pain still persisted. Clinical examination showed abdominal distention, no peristalsis, no signs of peritoneal irritation. A diagnostic laparoscopy to exclude possible progression of bowel ischemia and recurrent thrombosis, and also in order differentiate with other possible reasons of abdominal pain, was performed.

**Fig. 4:**
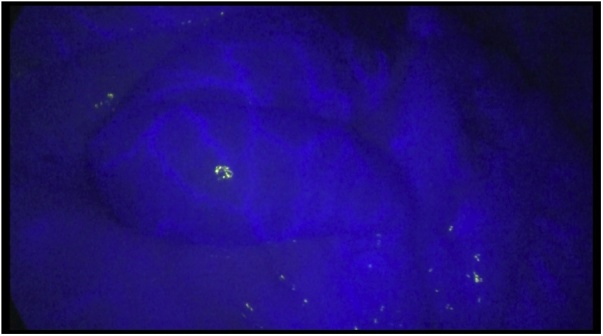
Diagnostic laparoscopy with indocyanine green imaging showing satisfactory bowel perfusion.

The operation revealed the dilated small bowel, but there seemed to be no necrotic bowel macroscopically. To confirm adequate bowel perfusion and viability a 0.25 mg of ICG was injected. We observed the bowel using the OPAL1 System (Karl Storz) (Fig. 4). The entire intestine wall showed satisfactory fluorescence emission, indicating no ischemia and necrotic changes. No other pathological changes in the abdominal cavity were detected.

The patient tolerated the surgery well and was transferred to the intensive care unit where, together with cardiologists, we carried out infusion, cardioprotective, detoxification and antibiotic therapy. On the day 6 after SMA stenting (9th in-hospital day), the patient was prescribed enteral nutrition. On the 15th day the patient was discharged in a good state of health with medical recommendations. We had a confirmation that he was alive at 4 months after the operative treatment of AMI.

## 3 Discussion

In patients with myocardial infarction, atrial fibrillation, accompanied by any invasive procedure, especially endovascular, the incidence of thrombosis and thromboembolic complications is high. This factor is the key to the suspicion of such a serious complication as AMI in the case of abdominal pain syndrome. In the event of this suspicion, it is necessary to perform a CT angiography in order to prevent the occurrence of AMI and also to exclude other causes of acute abdominal pain. This diagnostic measure has a sensitivity of 96% and specificity of 94% and reveals additional findings of the disease process, such as bowel wall thickening, dilatation, mesenteric fat stranding, pneumatosis, and portal venous gas [10].

Given the lack of robust collateral arterial splanchnic circulation, patients with SMA thrombosis rapidly progress in severity and often with infarcted small and large bowel; their mortality reaches 70–80% [3,5]. In case of timely diagnosed and no suspicion of bowel necrosis, endovascular techniques might be a first-line therapy for AMI [1]. Compared to open approach, they demonstrate significantly less overall complications [11,12].

In this case report we managed to perform angiography, endovascular thrombus extraction, SMA angioplasty with stenting within 8 h after the onset of pain. This time span allowed avoiding intestinal ischemia and necrobiotic changes. In this category of patients, the systemic inflammatory response syndrome may persist even after the restoration of perfusion, which does not exclude the development of intestinal wall complications, and requires diagnostic laparoscopy. Diagnostic laparoscopy allows evaluating not only the color and motility of the intestines, but also partly the pulsation of the mesentery vessels. At the same time, the ICG technology reveals greater possibilities. ICG fluorescence imaging captures the fluorescence of indocyanine green injected into the body using a digital video camera. Indocyanine becomes excited by infrared light and emits infrared fluorescence (peak wavelength, 830 nm) that can easily transmit through about 10 mm of human soft tissue [13]. Intravenously injected ICG is transported to peripheral vessels within a few seconds. In a tissue or an organ where blood flow is inhibited, the fluorescence emission of ICG weakens. The evaluation of blood flow using ICG fluorescence imaging is applied to breast reconstruction, coronary artery bypass grafting, and colorectal resection. This technique makes it possible to objectively assess the quality of tissue perfusion, which is indispensable when deciding on the viability of the intestine after AMI.

It must be assumed that in some cases, with adequate revascularization of the small intestine after thrombosis of SMA, reperfusion syndrome may occur. Reperfusion syndrome itself can cause ischemic necrosis of the intestinal wall. In such a situation, with an angiographic picture of adequate revascularization, data on ongoing necrobiotic phenomena might be missed. In this case, it is necessary to perform control laparoscopy within 24–48 h after blood flow restoration depending on the clinical condition of the patient to assess the quality of bowel perfusion and viability [1].

## 4 Conclusion

In patients with suspected AMI timely applying of MDCT, angiography, endovascular revascularization and ICG quality control of perfusion after revascularization are expedient to improve the results of treatment. These patients should be treated by a multidisciplinary team consisting of a cardiologist, a cardiovascular and endovascular surgeon, a general surgeon with experience in working with such patients. The development of endovascular techniques allows executing adequate thrombextraction and stenting in case of AMI in SMA system. The ICG imaging is an informative method for determining the quality of intestinal perfusion after revascularization and for objective assessment of the viability of the intestine.

## Sources of funding

None.

## Ethical approval

Ethical approval has been exempted by our institution, NGHCI Railway Clinical Hospital at the “Rostov-Glavnyy” station, OAO Russian railways, Rostov-on-Don, Russian Federation.

## Consent

Written informed consent was obtained from the patient for publication of this case report and accompanying images. A copy of the written consent is available for review by the Editor-in-Chief of this journal on request.

## Author contribution

A. Khitaryan – conceptualization, funding acquisition, investigation, methodology, project administration, resources, supervision, verification, writing original draft, writing review & editing.

I. Miziev I. – data curation, formal analysis, software, visualization, writing original draft, writing review & editing.

A. Murlychev – data curation, investigation, writing original draft, writing review & editing.

I. Taranov – data curation, investigation, writing original draft, writing review & editing.

O. Voronova – conceptualization, writing original draft, writing review & editing.

D. Shatov – conceptualization, writing original draft, writing review & editing.

A. Golovina – conceptualization, formal analysis, project administration, resources, software, visualization, writing original draft, writing review & editing.

D. Melnikov – conceptualization, writing original draft, writing review & editing.

## Registration of research studies

None.

This publication is neither ‘first-in-man study’ nor a research.

## Guarantor

Khitaryan А.G.

Golovina A.A.

## Provenance and peer review

Not commissioned, externally peer-reviewed.

## Declaration of Competing Interest

The authors declare no conflicts of interest. The authors have no financial, consultative, institutional and other relationships that might lead to bias or conflict of interest.
